# Supplementary material for: Are carbon emissions trading and green financial instruments synergistic? -Comprehensive quantitative research based on content analysis
Source: PLoS One. 2024 Mar 7;19(3):e0298601. doi: 10.1371/journal.pone.0298601 (PMC10919601; doi:10.1371/journal.pone.0298601)
Supplement: S1 Data — (ZIP) [file pone.0298601.s001.zip › Data/地级市Z_F工作报告.zip!/║╙▒▒/▒ú╢¿╩╨/└┤╫╘╣1⁄2╓┌║┼í╛┬φ┐╦╩2╛▌═°í┐_2018.docx]

2017年工作回顾

2月10日，市长郭建英在市十五届人大三次会议上作政府工作报告时说，2017年，是保定发展史上具有里程碑意义的一年。从市、县领导班子换届，到党中央、国务院决定设立雄安新区，再到党的十九大胜利召开，大事不断，喜事连连。一年来，在党中央国务院、省委省政府和市委的坚强领导下，我们以习近平新时代中国特色社会主义思想为指导，坚持新发展理念，抓住京津冀协同发展和设立雄安新区的重大历史机遇，高起点定位、深层次谋划、全方位推进工作，全市经济运行“总体平稳、稳中有升”，较好完成了市十五届人大一次会议确定的目标任务。全市生产总值完成3227.3亿元、增长6.0%（不含雄安增长7.3%，全省第一），一般公共预算收入完成238.4亿元、同比增长7.4%（不含雄安增长8.4%、全省第五），固定资产投资完成2581.9亿元（不含雄安增长6.6%、全省第三），实际利用外资增长8.7%（不含雄安增长16.2%、全省第二），社会消费品零售总额增长10.8%，城乡居民人均可支配收入分别增长8.5%、10%。一年来，全市上下团结一心，围绕打基础、增后劲，聚焦实干实政，做出了不懈努力：

以协同发展为统揽，全力服务雄安，主动对接京津，京津保率先联动发展势头更加强劲。聚焦雄安新区千年大计、国家大事，我们坚决服从、坚决支持、坚决借势、坚决保障，超前制定预案，加强综合管控，保障了雄安新区规划建设顺利起步。高标准启动新一轮规划编制，推动与雄安新区统筹、协调、错位、融合、一体化发展。深度推进与京津全方位对接合作，中电科涞水产业园、中船重工涿州海洋装备产业园、北京公交集团公交智造产业园、京投轨道交通产业园等一批重大项目签约落地，目前全市与京津合作项目436项、引资1800亿元。成功举办京津冀协同发展院士专家行、千人计划专家行、2017年“创响中国”（保定站）等大型活动。各方资源汇聚保定，社会各界看好保定，保定的战略地位和城市价值实现新提升。

着力打造协同创新共同体，用好中国科协、中关村、深圳三大创新发动机，引领高端发展，创新活力持续迸发。石保廊全面创新改革试验区建设走在全省前列，海外离岸人才创新创业基地、外国高层次人才来保工作许可等10项改革事项，被纳入省重点改革试验试点，京南国家科技成果转移转化示范区建设扎实推进。保定·中关村创新中心入驻企业105家，“一中心一基地多园区”发展格局基本形成，集聚效应、裂变效应不断放大。深保科创产业园开工建设，签约入园企业43家、意向入园企业96家，深安智谷、深圳光启、华讯方舟等一批“深字头”高科技平台和企业落户我市。与省科技厅建立厅市科技会商合作机制，保定高新区、河北农大入选第二批国家“双创”示范基地。全市现有高新技术企业433家、科技型中小企业6545家、院士工作站27家，居全省前列。

坚定不移调结构促升级，持续推进提质增效，经济发展质量不断提升。在京津冀地区率先通过“中国制造2025”试点示范城市评审，“253”制造业产业体系发展路线图进一步明晰。实施市以上重点项目350项、总投资9671亿元，其中省重点项目36项、总投资2022亿元。老工业企业退城搬迁改造按计划推进，我市钢铁产能全部退出。与百度合作共建智能交通示范城市，全国首个人工智能汽车双创基地——百度保定智能汽车研发中心落成，高碑店新凯无人驾驶通勤车在天津投入使用。航天乐凯新材料工业园、北大未名通天河生物产业园等重大项目加快建设，新材料、新一代信息技术、绿色建筑节能、航空航天等产业加快发展。高新技术产业、装备制造业增加值占规上工业比重分别达到44.6%、42.6%，高于全省26.2和17.5个百分点。农业现代化步伐加快，市级以上现代农业园区突破百个，数量、规模连续三年全省第一，农业产业化经营率预计68.8%。成功举办全市首届旅发大会，走出一条旅游资源匮乏地区发展旅游产业的新路。白沟采购贸易方式试点出口交易额突破5亿美元。开通中欧、中亚两条班列，年货物吞吐量150万吨，保定借势“一带一路”发展的前景可期。物流业高速发展，快递业务量突破2.5亿件。金融机构新增9家，全市各项贷款余额3406.6亿元、同比增长18.2%，增量存贷比95.2%、提高36.6个百分点。新增挂牌上市企业15家、达到96家，累计直接融资848亿元。各类市场主体60.4万户、增长13.6%，新注册企业3.9万户、增长14.41%。2017年中国百强城市排行榜我市列第59位，居全省第三。

持续打好污染治理组合拳，综合施策，科学防治，环境整治和生态建设成效显现。采取严格控煤举措，铺设燃气管网近2万公里，完成72.6万户气（电）代煤，全市10蒸吨以下、主城区35蒸吨以下燃煤锅炉淘汰改造实现“清零”，减少用煤260多万吨。主城区集中供热改造全部完成，铺设供热管网81.45公里，改造供热面积605.6万平方米，清洁供热率由2014年的39%提高到100%。整治散乱污企业2.4万家，重点整治了377家挥发性有机物排放企业。PM2.5浓度较2013年下降37.8%，全年优良天数159天，比2013年增加85天，空气质量达到近五年最好。去年秋冬季以来，PM2.5浓度同比下降44.76%，下降率在京津冀及周边“2＋26”个城市中排名第四，蓝天已不是稀客。统筹推进“绿满太行”行动和山水林田湖草整体修复，完成造林66万亩，森林覆盖率达到30.7%。全面推行“河长制”，实施白洋淀上游综合治理工程，水环境持续改善。严厉打击破坏环境行为，破获污染环境案件1943起，抓获犯罪嫌疑人2161名。

推进城乡统筹发展，攻坚克难理秩序，主动担当解遗难，城乡面貌不断改善。荣乌、西阜、涞曲、曲港、津石高速公路加快建设，廊涿城际、京雄石城际和保忻、保张、保沧铁路前期工作加快推进。集中开展拆违专项行动，拆除一批安全隐患大、顶风突击新建、妨碍重点项目、社会影响恶劣的违法建设，总面积达750多万平方米，震慑效应初步形成。下大力解决房地产开发遗留问题，436个项目、18.37万户“办证难”得以化解。探索创新住宅用地“双限双竞”出让模式，实施了限购、限贷、限房价、限地价等一系列调控措施，房价得到有效稳控。整改违法占地3.83万亩，矿山管控和深度治理取得积极进展。徐水区创造的国土管理新机制得到国土资源部肯定，并在我市召开的全国土地管理现场会上予以推广。我市被列为全国第三批“城市双修”试点城市，清河道署和淮军公所一期古城保护和修复项目完成。实施“七个一”“一补三化”等重点工程项目1079个、投资1793亿元，县城面貌明显改观。创建培育省级特色小镇12个，建成省级美丽乡村重点村418个，农村人居环境和生态环境不断改善。

持续推进共享发展，全力以赴打好脱贫攻坚战，社会事业不断进步，人民生活越来越好。严格落实“六个精准”“五个一批”，扎实推进五大精准脱贫工程，让惠民政策流到户里、洇进地里，望都率先脱贫摘帽，博野通过省级验收，涞水、易县达到脱贫标准，全市15万人实现脱贫。涞水旅游扶贫经验被列为中央政治局集体学习参阅资料，我市产业扶贫、金融扶贫做法在全国推广。统筹推进各项社会事业发展，莲池区、竞秀区等7个县（市、区）通过国家义务教育均衡发展评估。北京儿童医院保定医院迁入新址，第一中心医院门诊综合楼正式启用。城市公立医院改革扎实推进，药品加成全部取消。二孩政策全面落实。我市运动员在第十三届全运会上获得奖牌19枚，占全省三分之一。完成河北大学体育场建设，成功举办了中国足球甲级联赛。曲阳基层民政服务能力建设改革经验得到民政部肯定。我市宗教工作典型经验在全国推广。安全生产形势持续稳定，食品药品安全平稳向好。成功打赢H7N9防疫战，创造了重症病例救治奇迹。妥善处置黄金佳等非法集资案，圆满完成“5·23”“5·31”事故、阜平森林火灾、荣乌高速隧道塌方等急难险重任务。深入开展百日会战专项行动，扎实推进打防管控，破获刑事案件12923起、刑拘犯罪嫌疑人7583名，打掉犯罪团伙297个。坚决当好首都政治“护城河”，十九大安保任务顺利完成。“书香保定·香飘四季”全民阅读活动全省推广，社会主义核心价值观深入人心，成功创建省级文明城市。

注重加强政府自身建设，深化改革激发活力，转变作风提高效能，依法行政能力不断提升。市级机关内设机构改革基本完成，城市管理综合执法局、市县两级行政审批局组建运行。“放管服”改革不断深化，“两个代办”升级版全面推行，37个市直部门395项审批事项实现网上办理,全省“多证合一、一照一码”商事登记制度改革现场会在我市召开。“只跑一次”清单试点和“减证便民”行动在中央媒体重点推介。国地税征管体制改革稳步推进。国企改革“三供一业”移交地方按计划完成。自觉接受人大法律监督和政协民主监督，办理人大代表建议106件、政协提案448件，办复率100%。主动接受社会监督和舆论监督，受理群众来信来电、网络留言16万余件（条），办复率100%。推进党风廉政建设，做到有腐必反、有贪必肃，全年立案6018件，处分7187人，反腐败斗争压倒性态势已经形成。

过去的一年，我们以迎接党的十九大召开、深入学习宣传贯彻党的十九大精神为主线，准确把握保定所处的历史方位，确立了建设新时代新保定的奋斗目标，凝聚了推动京津保地区率先联动发展的强大合力。我们牢牢抓住协同发展重大机遇，借势雄安新区，锐意进取，扎实修为，夯实了保定跨越、崛起和复兴的发展基础。我们积极探索治霾治污的有效路径，推进城乡居民能源消费和生产生活方式变革，在广大群众充分理解、主动配合、大力支持下，完成了看似难以完成的北部九县和城市周边70多万户煤改气改电工程，为打赢蓝天保卫战提供了强大支撑。我们攻坚克难推动城市建设，妥善处置了一大批事关群众利益的疑难杂症和多年遗留问题，城市管理秩序进一步理顺。我们牢记总书记嘱托，坚持以人民为中心的发展思想，全力以赴打好脱贫攻坚战，广大群众的日子一天比一天好。

2018年目标任务和重点工作

郭建英说，党的十九大描绘了新时代中国特色社会主义现代化建设的宏伟蓝图。未来三年是决胜全面建成小康社会的关键时期，必须“强势起步、实干开路”，推动保定实现更好更快更大发展。我们要用三年时间，以造福人民为最大政绩，精准施策，对症下药，坚决打赢脱贫攻坚战，确保与全国一道同步迈入小康；以改善生态环境质量为着力点，持续用力，铁腕治污，坚决打赢蓝天保卫战，空气质量综合指数退出全国重点城市倒十名；以提高发展质量和效益为中心，创建“中国制造2025”国家级示范区，促进产业转型升级，建设现代化产业体系；以不断提升城市品位和人民生活质量为目标，把保定创建成全国文明城市，让人民群众在共建共享中增强获得感、幸福感；大力实施乡村振兴战略，开展农村人居环境整治攻坚行动，建设美丽乡村，推进城乡一体化发展。实现这些目标，我们必须咬定青山不放松，驰而不息，久久为功，一步一步向着目标迈进。

今后工作中，我们要走出一条质量更高、效益更好、结构更优、优势充分释放的发展新路，必须坚定不移维护以习近平同志为核心的党中央权威和集中统一领导，树牢“四个意识”，坚定“四个自信”，始终坚持用习近平新时代中国特色社会主义思想武装头脑、指导实践、推动工作；必须把握高质量发展根本要求，贯彻落实新发展理念，深化供给侧结构性改革，推动质量变革、效率变革、动力变革，努力实现更高质量、更有效率、更可持续的发展；必须抓住京津冀协同发展这个头等大事，借势雄安新区，做优环境，做大平台，打造发展高地，持续迈出京津保率先联动发展的坚实步伐；必须咬定既有战略部署不放松，突出一个“实”字，落实一个“干”字，矢志不渝、持续用力，一张蓝图干到底、不达目的不罢休；必须坚持以人民为中心的发展思想，始终把人民群众放在心中最高位置，心思围绕百姓转，事情按照民意干，用人民群众的获得感、幸福感、安全感来检验发展质量，努力让人民群众的生活更加美好！

2018年政府工作总的指导思想是：坚持以习近平新时代中国特色社会主义思想为统领，认真贯彻落实党的十九大和中央、省、市系列会议精神，坚持稳中求进工作总基调，按照高质量发展要求，统筹推进稳增长、促改革、调结构、惠民生、防风险各项工作，继续推进协同发展、创新驱动、环境支撑三大主体战略，持续打好城市做大、产业做强、生态做美、民生做富四场硬仗，全面加快与雄安新区联动发展，建设创新驱动发展强市，奋力夺取决胜全面建成小康社会的新胜利，开创新时代推动京津保地区率先联动发展的新局面。

经济社会发展的主要预期目标是：生产总值增长7%以上，一般公共预算收入可比增长9%，规模以上工业增加值增长5%，固定资产投资增长8%，社会消费品零售总额增长10%，出口总值增长6%，实际利用外资增长6%，服务业增加值增长10%以上，城乡居民人均可支配收入分别增长8%和9%，研发经费支出占GDP比重1.9%，PM2.5平均浓度下降完成省定任务，城镇新增就业9.5万人，居民消费价格涨幅控制在3%左右。

今年，紧紧围绕推动高质量发展，重点抓好八方面工作：

（一）持续推进协同创新，借势雄安建设，增强高质量发展动能。紧紧抓住协同发展这条主线，打造北京非首都功能疏解的支撑点、京津产业转移的承接地、外资进入京津乃至北方市场的桥头堡，在服务中借势，在创新中实现与雄安新区的联动发展。

深入推进精准对接。对标雄安新区，高起点高标准推进城市总规和各项规划编制。加快与雄安新区各领域对接，同步谋划推进交通设施、水利防洪设施和造林绿化建设，加快与雄安新区统筹、协调、错位、融合、一体化发展。健全与京津沟通对接机制，深化与科技部、中国科协、中科院、北大、清华合作，加强与央企、知名民企、世界500强对接，坚持定期互访、重点事项会商、互派干部交流，推动重点领域、重点项目和重要事项落地。

加快交通互联互通。对接雄安新区交通路网规划，开工建设廊涿城际、京雄城际铁路，推进雄保忻、保张、保沧铁路前期工作，确保太行山高速西阜和涞曲段、曲港高速、荣乌高速涞源段贯通，加快津石、京雄、荣乌新线等高速公路建设，推进京白线等断头路、容城—易县和安新—满城高等级公路和保定“大外环”建设，提升保定的全国性综合交通枢纽地位。

做强项目承接平台。围绕构建京津冀“2＋4＋N”产业合作格局，提升承载能力，抓好高新区、涿州国家农业高新技术产业开发区、中关村创新中心等一批重点承接平台建设，积极融入北京新机场临空经济区。加快推进中船重工、满城京投基地、博野三棵树等重大项目建设，完善重点项目双周调度会和现场办公制度，破解土地、人才、资金等瓶颈，推动重点项目早建设、早投产、早见效。

构建协同创新共同体。加快石保廊全面创新改革试验区和京南国家科技成果转移转化示范区建设，创建国家创新型城市。加强与中关村深度合作，推进满城中关村生命科学园、中关村保定·朝阳产业园、中关村高碑店软件园等项目建设。抓好与深圳合作，加快深保科创产业园、深安智谷、华讯方舟、深圳光启等重大项目建设。培育壮大创新主体，持续推进高新技术企业、科技型中小企业、上市公司“三个倍增”计划，全年新增高新技术企业60家以上、科技型中小企业500家以上。做大中电科涞水产业园、京投轨道交通产业园、高碑店国家建筑节能技术国际创新园等重点园区。实施创新平台升级行动，建设一批工程技术研究中心、企业技术中心、重点实验室等研发机构，新增院士工作站3家、创新平台10家。实施众创空间、科技企业孵化器倍增计划，催生一批科技小微企业。推进人才兴保工程，引进高层次创新创业团队，支持大学生创新创业。实施县域创新发展跃升工程，开展争创省级创新型县（市、区）活动。支持创新、鼓励创业，让新技术新产业新模式新业态层出不穷。

（二）持续加快转型升级，振兴实体经济，做强产业发展支撑。以提高供给体系质量为主攻方向，以产业转型升级为突破，构建“253”新型制造业体系，争创“中国制造2025”国家级示范区，建设现代化产业体系。

做大做强支柱产业。强化工业强市理念，加快发展先进制造业。汽车及零部件产业，大力发展新能源汽车、智能网联（百度）、自动驾驶汽车等，重点抓好大王店汽车及零部件产业园、立中清苑工业园、顺平汽车零部件工业园、涞水北京公交智造产业园、百度·保定智能汽车研发中心等项目，支持长城、立中、凌云等企业开拓国内国际市场，打造具有新内涵的“保定·中国汽车城”。新能源及输变电装备产业，依托英利、国电联合动力、四方三伊等企业，重点发展能源互联网和智能电网，抓好荣毅通信光电网、英利分布式光伏及微电网应用、宇能物理储能和水提氢发电、三川电力智能电网等项目，重塑“保定·中国电谷”品牌。

培育壮大新兴产业。启动战略性新兴产业三年行动计划，加快关键技术创新和成果转化，推动产业集聚和信息融合。高端装备制造产业，重点抓好标正高档数控机床、沈阳机床智能制造装备、科普菲尔中德智能制造、中船海洋装备、中航航天装备等项目。新材料产业，加快乐凯膜材料、光启超材料、同光晶体碳化硅、中创燕园氮化镓、邦泰氨纶、隆泰丰博石墨烯、立中隆达铝材料等项目建设。新一代信息技术产业，集中打造中科院第三代半导体检测中心、华讯方舟太赫兹基地、中国信息通信研究院检测基地和中电科涞水产业园、中印信息产业园、浪潮涿州大数据、蠡县高新电子产业园等项目。生物医药产业，重点发展现代中药、生物制药等，加快安国“数字中药都”、唐县北大未名通天河生物产业园、深圳北科生物基因治疗应用项目建设。节能环保产业，重点发展节能环保装备、资源循环利用技术、超低能耗建筑产业。军民融合产业，加快与军工央企合作，推进军民科技成果双向转化和共享，推进唐县、蠡县等军民融合产业示范园区（基地）和易县军民融合雷达产业园等项目建设，创建京津冀军民融合创新示范区，培育一批军民融合特色产业集群。

优化升级传统产业。通过引入新技术，开展新设计，全面提升传统产业层次和核心竞争力。纺织服装产业，加快白沟箱包集聚区建设，推动高阳纺织的高端化、智能化、规模化、集约化发展。食品加工产业，重点打造环京津食品加工产业带，建设京津、雄安绿色食品加工供应基地。绿色建筑节能产业，推进高碑店“列车新城”被动房示范项目、晨阳水漆环保涂料、顺平远大装配式建筑、清苑绿色建材产业园、易县新型建材循环经济产业园和博野生态涂装城建设，带动超低能耗建筑产业跨越式发展。开展质量提升行动，发展工业设计，提升“保定制造”的品牌竞争力。

加快发展现代服务业。坚持把发展现代服务业作为结构调整的重要突破口，推进“互联网＋”“旅游＋”，拓宽领域、提升功能、创新业态。加快涞水、易县、涞源、阜平创建国家级全域旅游示范区建设，抓好清西陵、狼牙山5A级景区创建。办好第二届市旅发大会，高质量高标准抓好秀兰休闲度假区、大激店音乐文化小镇等重点观摩项目，持续放大旅发效应。深化与阿里巴巴、京东合作，推动农村电商发展，启动涞水、涞源国家电子商务进农村综合示范县建设。推进莲池区京津保公铁联运国际物流园区和高碑店、白沟、安国等地物流产业聚集区建设，打造具有全国影响力的特色商贸物流基地。加快快递物流中心、快递产业园区建设，推进“快递下乡”，完善快递网络，努力把我市建设成为国家二级快递枢纽城市。高标准发展健康养老服务产业，培育发展科技服务产业，加快发展信息服务产业，大力发展共享经济、数字经济、现代供应链等新业态新模式，培育新的经济增长点。

培育更多市场主体。毫不动摇鼓励支持民营经济发展，不折不扣落实各项扶持政策，开展“降费减负”专项行动，降低实体经济成本。全面实施市场准入负面清单制度，破除歧视性限制和各种隐性障碍。实行领导干部联系重点企业“五个一”机制，及时帮助解决实际困难。完善诚信体系，守信联合激励，失信联合惩戒，引导市场主体以信誉赢得效益。尊重、支持、爱护企业家，保护激发企业家精神，构建亲清新型政商关系。大力支持本土企业家热爱家乡、发展家乡、建设家乡，真诚欢迎外埠企业家来保创业，共享雄安机遇，共创美好未来！

（三）持续加强规划建设管理，提升城市品位，打造区域性中心城市。加快新老城区融合发展，加强与雄安新区联动发展，打开城市大建严管新局面。

拉大城市发展框架。坚持规划引领，开展城市设计，高标准编制新一轮城市总体规划（2017-2035）。推进“多规合一”，编制城市立体交通、轨道交通规划，做大城市规模。加强城市主次干道建设，完成乐凯大街南延、复兴路西延，开工建设七一路西延、西二环北延，谋划启动南三环、北三环西延、东三环北延、西三环南延、长城大街南延、裕华路东延、向阳大街和阳光大街北延，打开城市外向通道。抓好七一路、莲池大街、恒祥大街、玉兰大街、三丰路等主次干道改造升级，加快瑞祥大街北延和北部新城配套路网工程建设，年底前竣工通车，畅通城市交通“微循环”。启动东湖片区和西湖体育新城片区市政设施建设，将学校、医院、市场等公共服务逐步配建到位。完成东湖片区景观、关汉卿大剧院和博物馆建设。

建设生态宜居城市。以“城市双修”为引领，治理城市病，改善人居环境，转变城市发展方式。抓好市区二环三环、火车站西广场、京广铁路沿线等重要道路和城市节点绿化景观提升，启动府河水系综合治理提升工程，完成竞秀公园、东风公园升级改造。实施淮军公所、清河道署片区城市“双修”工程，延续历史文脉，彰显古城底蕴。推进北三环、东风路西延地下综合管廊建设。加快大世界商业城、菊胡同等片区改造。启动新的三年棚改攻坚，加快推进现有城镇棚户区、城中村改造和老旧小区改善。

推进城市精细化管理。推进智慧城市建设，探索建立科学有序的管理体制和运行模式，推动管理重心下移，充实城管执法队伍，整合责任分区，试行街长制，实现管理精细化。规范管理共享单车、共享汽车、网约出租车、巡游出租车，综合治理占道经营，加快城市背街小巷改造，努力缓解交通拥堵，让群众出行更加便利。加强公交、公园、公厕、便民市场、停车场建设，实施公交智能便捷支付，改造提升街角游园，将主城区公厕改为水冲和环保厕所，建设一批便民市场，提供更多便民服务。继续开展房地产开发遗留问题专项整治，规范开发建设行为，让违法者退出市场。持续开展拆违风暴行动，坚决打击违法占地、违法建设行为，把“零容忍”坚持到底。

（四）持续聚焦“三农”问题，实施乡村振兴战略，加快农业农村现代化。按照“产业兴旺、生态宜居、乡风文明、治理有效、生活富裕”的要求，制定出台乡村振兴战略规划，推进各项工作，让广大农民过上更加幸福美好的新生活。

加快建设现代农业。实施农业供给侧结构性改革三年行动计划，大力发展质量农业、科技农业、绿色农业、品牌农业。落实市县粮食安全责任制，巩固和提高粮食产能，藏粮于地、藏粮于技，确保粮食安全。发展设施农业，提高畜牧、蔬菜、果品质量。抓好现代农业园区，推广太行山农业创新驿站模式，加快田园综合体、生态农场建设，打造京津优质农产品供应基地。支持望都创建国家农村产业融合发展示范园。强化全过程质量安全监管，三年实现“农产品质量安全县”全覆盖。推进农业产业化，推广“公司＋合作社＋基地＋农户”模式，发展多种形式规模经营，培育种养大户、家庭农场、农民专业合作社等新型农业经营主体。拓展产业链条，发展休闲农业、乡村旅游等新产业新业态，加快一二三产融合发展，把农业的增值收益更多留在农村、留给农民。

改善农村人居环境。实施农村人居环境整治三年行动，大力开展村庄、庭院绿化，建设美丽乡村。建立环境卫生长效保洁机制，形成“户分类、村收集、镇运输、县处理”的城乡统筹垃圾处理模式，推进“厕所革命”，解决农村脏乱差问题。加快“四好农村路”建设，推进外通内联、通村畅乡，新建改扩建农村道路800公里，把农村公路建好、管好、护好、运营好。加快改善农村通信、电网、环保等基础设施条件，着力建设生态宜居的农村人居环境。巩固提升农村饮水安全，加强农田水利设施建设。突出乡村特色，注重文化传承，加强村庄风貌整治。实施平安乡村创建行动，创新乡村治理体系，推进城乡公共服务均衡发展，让农民群众得到更多实惠。

加快发展县域经济。把壮大县域经济作为实施乡村振兴战略的主战场，打造立县强市的“四梁八柱”。做大县城，扩大城市规模，完善城市功能，持续推进“七个一”工程，加快国家园林城市（县城）创建，每个县（市）新建一个城市综合体、一所高水平学校、一所高水平医院，促进产城教融合发展。完善城镇体系，推进定兴非遗小镇、清苑好梦林水、易县燕都古城等省级特色小镇建设，谋划一批市级特色小镇，形成县城——特色城镇——农村新型社区的城乡融合体，促进就地城镇化和就近市民化。做强产业，实施产业集群发展能力提升工程，培育一批特色鲜明、辐射范围广、发展潜力大的龙头企业，构建带动县域经济发展的产业体系。做实园区，加快配套基础设施建设，引进一批立县项目，提升园区经济实力，争取更多县进入全省30强。

加快推进农村改革。以处理好农民与土地关系为主线，深化农村土地制度改革，完成农村土地承包经营权确权登记颁证工作。落实承包地“三权”分置政策，真正让农户的承包权稳下去、经营权活起来。开展“房地一体”农村宅基地和集体建设用地确权登记发证。把农村住房改造与土地整合转换结合起来，探索盘活闲置农房和宅基地的办法，努力破解村庄“空心化”、集体经济“空壳村”、基层组织“空白村”等问题。深化农村集体产权制度改革，多途径发展壮大集体经济。支持农民工、高校毕业生、退役士兵等各方面人才返乡下乡创业。

（五）持续打好污染防治攻坚战，全面改善生态环境，提高人居环境质量。下更大决心、用更强措施，解决环境突出问题，一天一天地努力，一个难点一个难点地突破，让生态环境更宜人、更宜居、更美好。

打赢蓝天保卫战。推进大气污染综合治理“1＋18”专项方案落实，以清洁取暖、抑尘、治污为重点，实施三年作战计划，确保年年有变化、三年大见效。淘汰全市范围内35蒸吨以下燃煤锅炉，完成气代煤收尾工程，推广煤改电，抓好农村太阳能取暖试点，探索推进多种清洁供暖方式，从根本上改变能源消费结构。加快重污染企业退城搬迁改造，开展工业企业全面达标排放行动，彻底整治散乱污企业。加强扬尘污染、机动车尾气治理，严禁焚烧秸秆、垃圾，严控挥发性有机物排放。抓好能源项目建设，力促大唐热电九期开工，深能西北郊、京能涿州热电项目一期工程全部投产。千方百计保障气源，加快徐水大王店、清苑、定兴、高碑店、涞水、满城、望都天然气调峰储备库和气化点供站建设，建立清洁能源保障体系，有效解决冬季重污染天气问题，让群众享有更多的蓝天白云。

提升水土环境质量。加强水污染治理，落实“水十条”，抓好重点流域污染防治，专项整治各类纳污坑塘和农村河渠，推进畜禽养殖禁养区专项整治。加大地下水污染防治，尽快消除黑臭水体。加快推进污泥处理中心、鲁岗污水厂深度处理、雨污分流整治工程，改善水环境。深化“河长制”改革，出台一河一策治理方案。落实白洋淀上游流域治理“1＋10”方案，从截污治污和调水补水着手，以府河、孝义河、白沟河、沙河灌渠为重点，推进污水处理提标扩容、河道清淤等“十大工程”。谋划启动安各庄、龙门水库联通工程，实现瀑河水库常态化蓄水。抓好总投资100亿元的易县抽水蓄能电站项目。探索建立河道常态化补水机制，高效利用南水北调长江水，用足用好引黄济淀黄河水，优化配置地表水，力争劣Ⅴ类水体断面基本消除，白洋淀上游流域水环境明显改善。落实“土十条”，加强农业面源污染治理，开展土壤污染状况详查，抓好土壤污染治理与修复试点，依法搬迁或关闭对土壤造成污染的企业。

加强生态环境修复保护。统筹山水林田湖草系统治理，大规模开展国土绿化三年行动。推进太行山生态涵养区、京津保平原生态过渡带、水系林网和村镇绿美廊道建设，抓好三北防护林、太行山绿化、白洋淀上游湿地修复和规模化林场建设，加快漕河沿线综合治理和一亩泉万亩湿地森林公园建设，完成造林65万亩。大力发展林下经济和森林旅游。加大露天矿山综合整治，不达标的坚决不准生产。保护基本农田，集约节约利用土地，严守资源消耗上线、环境质量底线、生态保护红线，加强生态监管，严厉打击破坏环境的违法行为。

（六）持续打好精准脱贫攻坚战，下真功夫、啃硬骨头，确保小康路上不落一人。把脱贫攻坚作为重大政治任务和第一民生工程，把脱贫质量放在首位，聚焦再聚焦、精准再精准，确保全年7万人脱贫，顺平、唐县、曲阳脱贫摘帽。

打好关键硬仗。打好深度贫困地区脱贫硬仗，新增资金、项目、建设用地指标向深度贫困地区倾斜，特别关注贫困老年人、残疾人、重病患者等重点群体，深度贫困地区的基础设施、教育医疗救助等方面取得根本性进展。打好易地扶贫搬迁硬仗，完成3.6万人国定搬迁任务，加快省定1.6万人搬迁进度。推进安置小区和产业园区“两区同建”，让群众搬得出、留得住、能致富。打好产业就业扶贫硬仗，以现代农业园区为载体，发展特色农林产业，规划建设设施蔬菜、食用菌、中草药、林果、畜禽养殖、优质杂粮六大产业片区和产业带，吸纳更多贫困人口就业增收，实现贫困户全覆盖。打好基础设施和村庄面貌提升硬仗，用好专项资金，提升贫困村水电路讯医校建设水平，完成贫困户危房改造。开展“共享农（山）庄”试点，探索贫困乡村振兴新路径。

补齐脱贫短板。补齐动态管理短板，建立完善贫困人口动态管理机制，确保应纳尽纳、应退尽退，保证贫困人口在动态中保持精准。补齐集体收入短板，所有贫困村实现有脱贫主导产业、有资产收益平台、有股份合作组织、有集体增收项目“四有”目标。补齐政策落地短板，试行医疗教育救助“一卡通”，打通服务群众的“最后一公里”。全面落实各项教育资助政策，筹集资金1.8亿元，资助贫困生15万人。补齐内生动力不足短板，坚持扶贫与扶志、扶智相结合，持续开展扶贫政策宣讲、技能培训、典型观摩活动，提高贫困群众自我发展能力。

完善长效机制。完善利益联结机制，推广“互联网＋扶贫”新模式，把贫困户和龙头企业、农民合作社、电商平台联为一体，让贫困户稳定分享产业增值收益。完善包扶帮扶机制，继续实施“五包一”“三包一”“一包一”定点帮扶，重点抓好北京帮扶的177个项目，深入开展民营企业“百企帮百村”行动。建立健全脱贫成果巩固提升机制，通过政策延续、产业增收、基金保险托底，确保脱贫群众持续稳定脱贫。加大教育扶贫力度，探索贫困家庭大学生就业安置政策，力促一人就业全家脱贫。完善低保制度和扶贫政策有效衔接机制，做到应扶尽扶、应保尽保。健全监督检查机制，强化项目资金监管，做到常态化督查全覆盖。

（七）持续深化改革扩大开放，聚焦问题破解瓶颈，释放发展活力。改革是活力之源，开放是进步之梯。进一步打开思想闸门，把改革步伐迈得更大，把开放大门打得更开。

深化重点领域改革。深入落实中央、省委系列改革任务，确保接得住、能生根、见实效。抓好石保廊全面创新改革试验，深化科技体制改革，推进海外人才离岸创新创业、高等院校岗位评价及“双师型”队伍建设改革。深化国企改革，加强国有企业信息披露、经营业绩考核、巡察稽查和国资监管。加快国有企业混合所有制改革，盘活存量资产。妥善处置历史遗留问题，完成“三供一业”改革和社区管理、医疗、教育等分离移交工作。深化“三医联动”改革，完善城乡居民基本医疗保障制度和大病保险制度，有序发展医联体，推进分级诊疗，提高基层医疗机构服务能力。深化财税金融改革，稳妥推进水资源税、环保税改革，推进农村信用社和城市商业银行改革。

进一步优化营商环境。以简政、减税、减费为重点，深化“放管服”改革，推动“多证合一”扩容升级，大力推行“双随机一公开”监管；深化行政审批改革，加快审批事项和人员划转，放大“一枚印章管审批”效应；规范公共资源交易运行，打造规范高效、公平公正的交易平台，以权力减法、服务加法，激发市场乘法。开展服务窗口质量提升行动，推进“互联网＋政务服务”，加快政府审批便企便民系统的互联互通，让信息多跑路，让百姓少跑腿，让群众和企业办事多点顺心、少点烦心。

积极扩大对外开放。积极推进“京津转移、保定承接，深圳北移、保定落地，雄安引领、保定支撑”战略，重点对接深圳、北京中关村，积极对接上海张江、武汉东湖新区，加强与央企、世界500强等战略投资者的对接合作，围绕“253”新型制造业体系，强化精准招商、产业招商、代理招商，促成一批高科技重大产业项目落地。以各类开发区为经济发展主阵地、项目建设主战场，打造一批中外合作产业园，积极推进高碑店国家建筑节能技术国际创新园、中关村中德智能制造产业园国际项目引进，加快大王店经济开发区国际汽配园建设。加强与中建、中冶、京投、北控水务、河北建设等企业集团的战略合作，加快基础设施建设步伐。支持汽车、新能源、纺织服装等优势企业走出去，扶持外贸综合服务企业扩大外贸出口。深化白沟市场采购贸易方式试点，加快建设保定中欧国际物流园，培育跨境电商，支持企业在中东欧、中亚、南亚设立海外仓，推进海外半成品加工基地、农产品生产基地和进出口商品展销基地建设，打造“一带一路”保定品牌。

防范化解金融风险。落实中央和省政策措施，加强地方金融类活动监管和风险排查，有效防控和处置银行不良资产。落实主体责任，严格控制、积极化解政府债务。加强小额贷款公司、融资担保公司、典当行等地方金融组织监管。坚决取缔非法金融机构，严厉打击违法违规金融活动，加大对圣瑞、黄金佳、e租宝等重大案件处置力度，落实防控责任，坚决守住不发生系统性、区域性金融风险底线。充分发挥金融保险的支撑作用，加强政银保企对接，鼓励创新产品和保险服务，大力支持实体经济发展。做好企业上市和债券融资，力争每个县新增一家挂牌上市企业。深化农村金融改革，继续推动农村信用社改制，推广阜平金融扶贫典型经验，拓宽农业投融资渠道。创优金融生态环境，提升金融服务实体经济能力。

（八）持续补齐民生短板，提供更多公共服务，增强人民获得感幸福感安全感。坚持以人民为中心的发展思想，围绕群众需求抓民生，一年接着一年干，不断满足人民群众的生活需要。

发展教育事业。实施中心城区学校建设三年攻坚行动，年内启动新建、改扩建学校30所。谋划启动县城学校建设三年攻坚行动，高标准规划建设一批县城中小学、幼儿园，努力解决中小学生课外负担重、“大校额”“大班额”问题。吸引社会力量举办托幼机构，规范整顿民办幼儿园办园行为。推进义务教育基本均衡发展，实现国家级评估验收全覆盖。加快推进保定职教园区建设。全面深化新时代教师队伍建设改革，加强师能师德师风建设，营造尊师重教浓厚氛围。

提升医疗水平。加快卫生项目建设，启动市第一中医院迁建、第一中心医院东院改扩建，完成市妇幼保健院迁建、市第二医院病房楼扩建、市第三中心医院改扩建工程并投入使用。创建河北省中医药强县，打造中医药强市，建设健康保定。鼓励社会资金进入养老、医疗领域，发展养老服务业，社区和居家养老服务设施覆盖80%的城市街道。

丰富文体生活。实施中华优秀传统文化传承发展工程，建立健全公共文化服务设施网络，推进电影下乡，启动市档案馆、图书馆建设，完成规划馆、博物馆展陈建设。加强体育设施建设，办好体育赛事，推动全民健身，活跃群众文体生活。做好市长专线工作，畅通百姓与政府的沟通渠道。推广社区、社会组织、专业社工三社联动模式，加强城乡社区治理。践行社会主义核心价值观，弘扬时代精神，推进社会公德、职业道德、家庭美德、个人品德建设，提高全社会文明素养，力争三年建成全国文明城市。

抓好社会保障。突出抓好农民工、城镇困难群体就业和退役军人安置工作，依法打击拖欠农民工工资行为。全面实施全民参保计划，城乡居民年低保标准分别提高到6600元和3700元，基本医保财政补助标准再提高40元，基础养老金最低标准再增加18元。落实医疗救助扶贫政策，完善社会救助体系，提高低保对象、特困人员、残疾人等保障标准。加快供热、供气管网互联互通，积极争取气源，保障群众温暖过冬。坚持“房子是用来住的、不是用来炒的”定位，加快建立多主体供应、多渠道保障、租购并举的住房制度，支持居民自住购房需求，抑制投资投机性购房，积极稳控房价，防止大起大落。

统筹其他事业。加强统计监管，开展第四次全国经济普查，继续做好广播电视、新闻出版、外事侨务、气象地震、防灾减灾、邮政通信、史志档案和老龄、妇女儿童、残疾人等工作。充分发挥工会、共青团、妇联等人民团体桥梁纽带作用。积极维护民族团结，全力促进宗教和谐。加强国防教育、国防动员和国防后备力量建设，支持驻保部队推进新时代强军事业，持续推进双拥共建，巩固军政军民团结的好局面。

建设平安保定。深入开展信访隐患排查，着力解决好房地产办证难入住难、食品药品安全、环境污染等群众反映强烈的突出问题，及时消除和化解社会不稳定因素。严格落实安全生产“一岗双责”，加强风险分级管控，实施安全事项清单管理，集中开展打非治违、城市燃气、建筑施工、道路交通等专项整治，严防重特大事故发生。强化食品药品全过程监管，确保人民群众饮食用药安全。开展扫黑除恶专项斗争，坚持依法严惩、打早打小、除恶务尽，始终保持对各类黑恶势力违法犯罪的严打高压态势；严厉打击涉枪涉爆等严重暴力犯罪和“两抢一盗”、网络诈骗等多发性侵财犯罪，妥善处置突发事件，建设平安保定。

在多渠道多层次征求群众意见的基础上，按照尽力而为、量力而行的原则，今年市委、市政府在教育、就业、社会保障等七个方面确定了47项惠民实事。我们一定坚持以民为先，精力、财力向实事倾斜，努力把实事办实、好事办好。

努力建设务实高效政府

郭建英说，走进新时代，开辟新征程，谱写新篇章，我们一定高举习近平新时代中国特色社会主义思想伟大旗帜，以时不我待、只争朝夕的精神，逢山开路、遇水搭桥，实干苦干拼命干，不断开创各项工作新局面。

（一）讲政治，增强本领，服务人民。学懂弄通做实党的十九大精神，牢固树立“四个意识”，扎实开展“不忘初心、牢记使命”主题教育，全面提升讲政治、识大局、定政策、促改革的能力。提高政治站位，牢记党对一切工作的领导，不折不扣地贯彻党中央决策部署。坚持全心全意为人民，凡是群众反映强烈的问题都要严肃认真对待，凡是损害群众利益的行为都要坚决纠正。营造良好的政治生态环境和宽松的政务环境，把难得机遇加速转化为发展动力，实现保定的跨越崛起和复兴。

（二）重法治，依法行政，阳光行政。把遵守宪法和法律作为施政的根本原则，加快法治政府建设。落实法律顾问制度，严格依法民主决策，严格规范行政权力，严格规范执法行为，自觉接受人大法律监督、政协民主监督和社会舆论监督。坚持“法无授权不可为、法定职权必须为”，不失位不越位不缺位，把经济管理权放到离市场最近的地方，把社会管理权放到离老百姓最近的地方。扎实开展普法宣传教育，领导干部必须带头尊法学法守法用法，让政府工作依法进行，让政府权力在阳光下运行，有权不任性，用最佳规范赢得人民群众的尊重。

(三)强担当，守土履责，尽职尽责。新时代领导干部的时代责任就是能干事、敢担当、有作为。勇于直面矛盾、勇于攻坚克难、勇于迎难而上，要成为政府机关的精神特质。在实际工作中多当“主攻手”，不当“二传手”，更不当无为无过的“木头人”，走到前边去“领”，深入一线去“导”，一级干给一级看，一级带着一级干，形成“头雁效应”。推行目标责任制，定性定量相结合，做好清单管理，不驰于空想，不骛于虚声，同向发力，把应做的工作做实着，把要办的事情办实诚，把急办的任务早干成。

（四）转作风，真抓真干，求实务实。深入开展“纠四风、转作风、抓落实”专项行动，深入开展“十查十改”，着力解决“表态多调门高、行动少落实差”“门好进、脸好看、事难办”“只让领导注意、不让群众满意”“不贪不占也不干”等突出问题。开展大学习、大调研，增强看问题的眼力、谋事情的脑力、察民情的听力、走基层的脚力。扑下身子，苦干实干，多干事、干实事，一步一个脚印，踏踏实实把工作做好。

（五）严管理，勤政廉政，清正清明。把纪律和规矩挺在前面，贯彻落实《准则》和《条例》，严格执行中央八项规定精神和《实施细则》。持之以恒正风肃纪，驰而不息反腐倡廉，着力解决审批监管、资源开发、金融信贷、扶贫攻坚等重点领域和关键环节的腐败问题，坚决查处违规违纪和失职渎职行为。坚决整治群众身边腐败问题，“老虎”“苍蝇”一起打，坚决查处涉黑“保护伞”。把践行“三严三实”贯穿于全部工作生活中，向廉洁自律的高标准看齐，做到心有所戒、行有所止，守住底线、不碰高压线。营造良好政治生态，努力建设一支信念坚定、为民服务、勤政务实、敢于担当、清正廉洁的公务员队伍。

新思想引领新时代，新使命开启新征程。让我们更加紧密地团结在以习近平同志为核心的党中央周围，在省委、省政府和市委的坚强领导下，高举习近平新时代中国特色社会主义思想伟大旗帜，深入贯彻落实党的十九大精神，不忘初心、牢记使命，同心同德、奋力向前，以更加昂扬的斗志、更加务实的作风，为决胜全面建成小康社会、谱写新时代新保定发展的新篇章而努力奋斗！
